# Optical Remote Sensing of Glacier Characteristics: A Review with Focus on the Himalaya

**DOI:** 10.3390/s8053355

**Published:** 2008-05-23

**Authors:** Adina E. Racoviteanu, Mark W. Williams, Roger G. Barry

**Affiliations:** 1 Department of Geography, University of Colorado, UCB 260, Boulder CO, 80309, USA; 2 Institute of Arctic and Alpine Research, University of Colorado, UCB 450, Boulder CO, 80309, USA; 3 National Snow and Ice Data Center, CIRES, University of Colorado, UCB 449, Boulder CO, 80309, USA

**Keywords:** remote sensing, ASTER, DEM, glaciers, mass balance, Himalaya

## Abstract

The increased availability of remote sensing platforms with appropriate spatial and temporal resolution, global coverage and low financial costs allows for fast, semi-automated, and cost-effective estimates of changes in glacier parameters over large areas. Remote sensing approaches allow for regular monitoring of the properties of alpine glaciers such as ice extent, terminus position, volume and surface elevation, from which glacier mass balance can be inferred. Such methods are particularly useful in remote areas with limited field-based glaciological measurements. This paper reviews advances in the use of visible and infrared remote sensing combined with field methods for estimating glacier parameters, with emphasis on volume/area changes and glacier mass balance. The focus is on the **A**dvanced **S**paceborne **T**hermal **E**mission and **R**eflection **R**adiometer (ASTER) sensor and its applicability for monitoring Himalayan glaciers. The methods reviewed are: volumetric changes inferred from digital elevation models (DEMs), glacier delineation algorithms from multi-spectral analysis, changes in glacier area at decadal time scales, and AAR/ELA methods used to calculate yearly mass balances. The current limitations and on-going challenges in using remote sensing for mapping characteristics of mountain glaciers also discussed, specifically in the context of the Himalaya.

## 1. Introduction

An increasing number of glaciologic studies are focusing on monitoring glacier changes using remote sensing in mountain regions experiencing rapid changes in glacier extents, such as Alaska [1], Patagonia [2], the Andes [3-6], the Alps [7-9], the Himalaya [10-16] and Central Asia [17-19]. Mass-balance records show an acceleration of glacial loss in the last decades in many of these sites [20, 21]. Such studies show the potential of remote sensing data to provide useful information for glaciologic applications such as: glacier area, length, surface elevation, surface flow fields, accumulation/ablation rates, albedo, equilibrium line altitude (ELA), accumulation area ratio (AAR) and the mass balance gradient δb/δz. In particular, the last three parameters are of importance for mass balance monitoring, as they react to annual fluctuations in climate parameters such as precipitation, temperature and air humidity. While recent trends of glacier retreat may be attributed due to 20^th^ century climate fluctuations, the response of glaciers to such climate fluctuations is complex, and may also depend on non-climatic factors such as ice dynamics, glacier hypsometry (the distribution of glacier area versus elevation) and topography [22].

Traditionally, glacier mass balance was measured with the “direct” glaciologic method [23-25], which consists of placing a network of stakes and pits at representative points on the glacier surface. Stakes are drilled into the ice and the distance between the top and the bottom of the stakes is measured. In the accumulation area, snow pits are dug, and the thickness of the accumulation layer is detected by changes in grain size or the presence of a layer of dirt. The thickness divided by the average density yields the specific winter mass balance at a point on the glacier surface. Measurements are conducted either between two fixed dates, or at the end of the accumulation and ablation seasons. Due to intense manual labor, this method has limited applicability in rugged or remote glacierized areas due to logistic difficulties involved in maintaining a monitoring network, lack of logistical support and political or cultural conflicts. In such areas, spaceborne remote sensing may offer complementary information on glacier parameters, especially glacier area, surface elevation, ELA and terminus position, from which mass balance can be inferred on various spatial and temporal scales. Scenes acquired at the end of the ablation season are useful to identify the end-of-summer snowline altitude (SLA), considered to be directly related to variations in a glacier's mass balance, and used as surrogate for ELA on temperate glaciers [26].

The increased availability of imagery from remote sensing platforms with adequate spatial and temporal resolution, near global coverage and low financial costs allow extending the measurements of glacier parameters over larger areas and longer time spans. There is a need for continuous monitoring of the properties of mountain glaciers in poorly surveyed glacierized ranges such as the Himalaya. While increasingly comprehensive measurements of glacier area have been made in many glacierized areas of the world since the 1960s [27], there remains a significant gap in Himalayan glacier research. With a few exceptions [11, 28-31], glacier monitoring in this area is limited to observations of glacier termini data. Himalayan glaciers are conspicuously absent from global mass balance records [21]. Existing glacier inventories are outdated and sparse. With a few exceptions, for example [32, 33], these inventories are not in the public domain or simply restricted, such as [34-36].

This paper reviews the potential of visible and thermal infrared remote sensing data combined with Geographic Information Systems (GIS) and field methods for estimating the characteristics of alpine glaciers, with a focus on the Himalaya. We describe the various steps involved in estimating these characteristics from satellite imagery, particularly ASTER data and discuss their applicability for Himalayan glaciers: 1) semi-automated glacier delineation algorithms from multi-spectral and topographic data; 2) glacier thickness and volume estimations from satellite data; 3) volumetric changes at decadal time scales using digital elevation models (DEMs) on a pixel by pixel basis and 4) AAR-ELA methods to calculate yearly mass balances of glaciers from multispectral data. The emphasis is on the advantages and limitations of remote sensing methods for mass balance estimations at various spatial and temporal scales, and their potential in filling the existing gap in mass balance records in the Himalaya.

## 2. Optical remote sensors for glacier monitoring

Components of mass balance (accumulation and ablation) cannot be measured directly from space [37], but parameters extracted from airborne and spaceborne scanning (glacier area, terminus position, transient snowlines and surface elevations) can be used to estimate glacier-wide mass balances. Until the early 70s, aerial photography was the primary remote sensing technique available for extracting glacier parameters. In many parts of the world, however, aerial photographs are restricted for political reasons, or simply not available due to the high costs of flying camera-equipped aircrafts. Medium-resolution satellite data (10 - 90 m) have become available for cryospheric studies since the early 1970s, with the launch of new spaceborne sensors: Landsat Multispectral Scanner (MSS), Landsat Thematic Mapper (TM) and Enhanced Thematic Mapper Plus (ETM+), System Pour l'Observatoire de la Terre (SPOT), Terra ASTER, the Indian Remote Sensing Satellite (IRS), and more recently the Advanced Land Observing Satellite (ALOS) launched in 2006. Other optical sensors with meter and sub-meter spatial resolution such as IKONOS, Quickbird and GeoEye-1 provide satellite imagery comparable to aerial photography, suitable for detailed glacier studies at basin scales. However, the high costs, narrow swath size (11 km for IKONOS and 16 km for Quickbird, respectively) and long revisit intervals of a few months limit their use at large spatial scales. Sub-meter imagery acquired from the American intelligence spy satellite series CORONA from 1960 to 1972 were declassified in 1995, and are available for some glacierized areas from the Earth Resources Observation and Science (EROS) (http://edc.usgs.gov).

Optical sensors detect solar radiation reflected by the earth's surface in the visible (VIS) and near infrared (NIR) bands of the electromagnetic spectrum (0.35 – 2.5 μm) and the radiation emitted by the surface in the thermal infra-red (TIR) (8 – 14 μm), recorded as brightness temperature by the sensor [38]. The capability of these sensors to acquire data at medium spatial resolutions of 10 m to 90m in multispectral mode, with relatively large swath widths (185 km for Lansdat, and 60 km for ASTER) and short revisit times (16 days for ASTER), makes them useful for regular glacier mapping over extensive areas. The thermal band of Landsat ETM+ (10.4– 12.5 μm, at 60 m pixel size) and the multispectral thermal bands of ASTER (8.125 – 11.65 μm, at 90m pixel size) may provide the potential for distinguishing debris-cover on glaciers [39]. Furthermore, the ASTER, SPOT5, IRS-1C, and CORONA KH-4, KH 4A and KH 4B have the capability of acquiring stereoscopic images, from which elevation data can be extracted for monitoring of the glacier surface in three dimensions.

The Advanced Land Observing Satellite (ALOS) launched on January 2006 by the Japanese Earth observing satellite program combines the advantages of visible remote sensing with active microwave techniques using three remote-sensing instruments: the Panchromatic Remote-Sensing Instrument for Stereo Mapping (ALOS PRISM), suitable for detailed digital elevation mapping; the Advanced Visible and Near Infrared Radiometer type 2 (AVNIR-2) suitable for glacier mapping in the visible and near infrared, and the Phased Array type L-band Synthetic Aperture Radar (PALSAR), suitable for day-and-night and all-weather land observation. The projected elevation extraction accuracy of ALOS PRISM is 5 m [40]. The AVNIR-2 sensor provides data on the albedo of the glacier surface, and is useful for mapping of the glacier area. The PALSAR multi-polarization and multi-incidence angle observation are promising for estimating snow cover depth, which may assist in determining accumulation rates on glaciers [40]. Data are available by request through ALOS Data Nodes (ADN) for non-commercial use at costs incurred by the participating ADN organizations by region, as specified in [40]. The potential of ALOS for glaciologic applications has been explored only by a few studies so far: [41, 42], mostly to complement results from other sensors. For example, [42] used ALOS/PRISM to validate ASTER/Landsat-derived outlines for benchmark glaciers in the Tien Shan. The development and validating of algorithms for extracting physical parameters, including DEMs generation and image orthorectification, are still tasks in progress at the Japan Aerospace Exploration Agency (JAXA).

Currently, ASTER may still be the most suitable sensor for monitoring of glacier parameters, including mass balance applications. Advantages over the other sensors include: (1) ASTER's spatial resolution of 15m in VNIR is adequate for regional-scale glacier studies; (2) the high spectral resolution with 3 VNIR bands, 6 mid-IR bands and 5 TIR bands allows for multi-spectral image classification (3) the off-nadir viewing band in the NIR enables high-resolution along-track stereoscopic vision and (4) the adjustable sensor gain settings provide increased contrast over bright areas (snow and glaciers) [43]. Repeated images are acquired every 16 days, with the possibility of increasing the frequency to two days in the event of natural disasters [44]. The suitability of a particular scene depends on: a) the presence/absence of seasonal or temporary snow; b) the percentage of cloud cover; c) the date of acquisition. Ideally the images used should be acquired at the end of the ablation season for minimal seasonal snow cover, with instrument gains customized for high contrast over the glaciers. ASTER-derived data are increasingly being used to update glacier parameters, and are available on a cost-free to regional centers for the Global Land and Ice Measurement from Space (GLIMS) project [44]. Various products are available from the Land Processes Distributed Active Archive Center (LP DAAC, http://edcdaac.usgs.gov): AST14DMO, the orthorectified product package based on L1B data (registered radiance at sensor), AST08 (surface kinetic temperature), AST05 (surface emissivity) and AST07 (surface reflectance) among others. The orthorectified product contains 14 ASTER bands and the “relative” DEM constructed on-demand from bands 3n and 3b using the Silcast software. As of February 2008, more than 180,000 ASTER images acquired over glaciers are stored in the GLIMS Glacier Database (http://www.glims.org). High priority data acquisition requests (DARs) submitted by the researcher to the Aster Science Team ensure adequate quality of the acquired data for glaciologic applications. DARs include specifications on instrument gain settings for each ASTER band, the acquisition window (start and end time for the acquisition), and specific glaciers to be targeted in the field [45].

A major disadvantage of the visible and near-IR (VNIR) sensors, including ASTER, is their limitation to daylight, cloud-free conditions, which are difficult to obtain over extensive glacierized areas such as the Himalaya. Active microwave systems such as Synthetic Aperture Radar (SAR) are efficient in cloud areas, but the severe geometric and radiometric distortions and speckle (“noise”) require complicated processing and accurate digital elevation models (DEMs), which are not always readily available [38]. Other techniques such as passive microwave systems, radar, and laser altimetry show promise for increasing our understanding of glacier characteristics in the Himalayas [46-49]. However, here we focus on optical sensors, because these sensors are already launched and are providing important sources of data.

## 3. Remote sensing methods for glacier monitoring

The traditional “glaciologic” method for determining glacier mass balance consists of placing a network of stakes and pits on the glacier surface and measuring the change in surface level (accumulation and ablation) while taking into account snow/firn density, either between two fixed dates (annual mass balance) or at the end of the ablation and accumulation seasons (seasonal mass balance) [24, 25]. The equilibrium line altitude (ELA) is the average altitude at which accumulation balances ablation over one year [50]. On an annual basis, the ELA reacts to a combination of climate variables, particularly precipitation and air temperature. The long-term average, or *steady-state* ELA, is the altitude for which the glacier as a whole has a mass balance of zero, and is said to be in equilibrium with climate. Whether the annual ELA is above or below the steady-state ELA is a key indicator of the state of health of a glacier: annual ELAs that are higher than the steady-state ELAs indicate a negative mass balance for that particular year.

In remote, rugged areas such as the Himalayas, the glaciologic method is difficult to apply due to complicated logistics and political or cultural conflicts. A new remote-sensing approach used to estimate glacier mass balance involves determining the total mass balance of a glacier from measured or inferred volumetrics and/or ELA altitudes. Other approaches exist, such as the “budget approach”, thoroughly reviewed in [37]. In this method, the change in local mass balance is inferred from ice thickness and glacier flow components. The parameters needed - ice thickness, surface and depth-average velocity and specific mass balance - however, are not easily determined from remote sensing, especially for alpine glaciers [37]. Thus, the budget approach has only been applied to Antarctica and Greenland [51]. Here we focus on direct measurements of area, thickness and ELA changes for alpine glaciers to infer mass balance, particularly: 1) volume-area scaling techniques; 2) AAR-ELA methods to calculate yearly mass balances; and 3) elevation changes using DEMs on a pixel by pixel basis. Accurate glacier outlines and DEMs are needed as input for volume and/or mass balance calculations. The following sections provide a brief discussion of the remote sensing mass balance methods, with a focus on the preliminary steps involved (glacier outline and DEM generation), and field validation. For detailed information, the reader is directed to [37] and a more recent review focusing on Patagonia ice fields [52].

### 3.1. Glacier ice delineation

Changes in glacier area and terminus positions have been used widely as indicators of a glacier's response to climate forcing [53]. These two parameters are relatively easy to extract from multispectral satellite images. Glacier area calculated from remote sensing outlines may be used as input for volume-area scaling techniques [54, 55]. When combined with a DEM, glacier outlines serve to derive glacier parameters such as hypsometry, minimum/median elevations and ELA [56, 57]. For example, a recent study used glacier outlines and a DEM for calculating glacier length fluctuations as direct indicators of changes in mass balance [58]. This section discusses commonly used band combination techniques for automatic delineation of glacier outlines from ASTER imagery, with emphasis on debris-cover mapping procedures, field validation and limitations of each procedure. An evaluation of other classification techniques for glaciers, including supervised classification techniques is provided by [7, 59], and the accuracy of these various classification methods was addressed by [60].

Automatic delineation of clean glacier ice relies on the spectral uniqueness of glacier ice in the visible and near-IR part of the electromagnetic spectrum (ASTER bands 1, 2 and 3). Snow and ice are characterized by: 1) highly reflectivity (albedo) in the visible wavelengths (0.4 – 0.7 μm); 2) medium reflectivity in the near-infrared (0.8 – 2.5 μm); 3) low reflectivity and high emissivity in the thermal infra-red (2.5 - 14 μm); and 4) low absorption and high scattering in the microwave [38]. The optical properties of snow are a function of the bulk properties of constituent ice grains, particulate impurities and liquid water [61]. The most important property is the imaginary part of the dielectric constant, which determines the degree of absorption. Ice is almost transparent in the visible (VIS) wavelengths (0.4 – 0.7 μm) where the reflectivity is insensitive to grain size but is sensitive to the amount of impurities. Ice is moderately absorptive in the near-infrared (NIR) (0.8 – 2.5 μm) and its absorption increases with wavelengths greater than 2.5 μm.

In clear weather, the high albedo of snow and ice make them easily distinguished from surrounding terrain using the VIR bands of ASTER (0.52 – 0.86 μm). Optically thick clouds are also highly reflective in the VIR, confounding the classification, but are discriminated from snow and ice in the 1.6 – 1.7 μm wavelengths (band 4 of ASTER). At these wavelengths, clouds are reflective but snow and ice are absorbing [62]. Commonly used techniques such as single band ratios and the normalized difference snow index (NDSI) [63] take advantage of the high brightness values of snow and ice in the visible wavelengths to separate them from darker areas such as rock, soil, or vegetation. The latter is similar to the Normalized Difference Vegetation Index (NDVI) used for vegetation mapping. NDSI is calculated as (VIS-SWIR) / (VIS+SWIR), where VIS is band 1 of ASTER (0.52 – 0.6 μm) at 15m and SWIR is band 4 of ASTER (1.6 – 1.7 μm) at 30m. Band 4 of ASTER needs to be resampled from 30m to match the spatial resolution of the visible bands (15m) for band ratio computations. The resulting NDSI image has values from -1 to 1 and is segmented using a threshold value to obtain a binary map of glacier – non-glacier areas. Pixels with NDSI values greater than the threshold value are assigned to snow/ice class, and those less than the threshold value are classified as non-snow or non-ice. An NDSI threshold of 0.4 was found to differentiate snow from non-snow by [63]. Thresholds of 0.5 - 0.6 proved successful in delineating glacier ice for the Peruvian Andes [3]. Another study [64] used NDSI in a supervised maximum-likelihood classification scheme based on principal components analysis, band ratios and a false color composite, which distinguished between snow, ice and debris cover. Glacier ice has a lower albedo than fresh snow, so the threshold needs to be adjusted accordingly by visual inspection. Single band ratios (VIS/NIR) such as ASTER 3/ASTER 4 were used by [7, 9, 65], with results similar to NDSI. However, NDSI has shown to produce a less noisy map and to remove some of the illumination effects present on glaciers, yielding satisfactory results in shaded ice [3].

Both NDSI and single band ratio classification techniques have the advantage of being fast and robust, and thus relatively easy to automate over extensive areas. However, some difficulties in automatic mapping of glaciers using band ratios remain due to: 1) the presence of pro-glacial and supra-glacier lakes; 2) the presence of fresh snow on the glacier surface; and 3) debris-cover on glaciers. Pro-glacial turbid lakes, frozen lakes and supra-glacial lakes are mis-classified as glacier because the bulk optical properties of liquid water are very similar to ice in the visible and near-infrared wavelengths [66]. Mapping of glacier lakes from satellite imagery is still not well developed, but two studies proposed new techniques using ASTER imagery [67, 68].

Probably the greatest difficulty in mapping glaciers from remote sensing is the presence of debris cover on glaciers. Glacier areas covered by debris confound the processing techniques presented above. Debris cover on glaciers has a similar VIS/NIR spectral signature to the surrounding moraines [69], due to similar reflectance at these wavelengths. Spectral information alone is insufficient for mapping ice covered by debris, and manual digitization is time-consuming and subject to human error. Combining band ratios with topographic information shows promise for semi-automated mapping of debris cover. For example, [69] used a slope map derived from a DEM, a false color composite of TM bands 3, 4 and 5 and a TM4/TM5 band ratio to map debris-covered ice in the Swiss Alps. A relatively new technique based on thermal takes advantage of the difference in the temperature of the debris overlaying ice versus the temperature of the surrounding moraines. Debris underlined by glacier ice is generally colder than the surrounding non-ice moraines if the debris is thin (< 2cm) [70, 71]. Above this “critical” threshold, ice is insulated because of the thermal conductivity of debris (0.56 W/m/K), considerably lower than that of ice at 0°C (2.10 W/m/K) [22]. The surface temperature of thick debris overlying glacier ice is similar to the surrounding moraines, confounding the signal [72]. For thin debris cover, however, this difference in temperature of debris cover on ice versus ice-free moraines results in different temperature brightness values recorded by the thermal bands of Landsat or ASTER. For example, [70] and [73] used surface temperature data derived from thermal IR images to estimate the debris thickness and thus infer the melt rate under a debris layer. Taschner and Ranzi [39] were able to distinguish debris cover on glaciers in the Italian Alps from the thermal bands of ASTER and Landsat TM. The applicability of the thermal approach alone is currently limited to thin debris cover, but shows promise when combined with multispectral data and morphometric information, as in [17]. However, manual input is still needed to develop a standardized semi-automated mapping algorithm.

### 3.2 Extracting glacier parameters from DEMs

Remote sensing-derived outlines combined with DEMs in a GIS provide glacier parameters such as length, termini elevations, median elevations, hypsometry maps and glacier flow patterns at different time steps. DEMs combined with glacier outlines are also useful to derive ice divides in a semi-automated fashion [3, 74]. A global elevation dataset was developed from the Shuttle Radar Topography Mission (SRTM), flown in February 2000 [75]. The “finished” elevation datasets with ∼90 m spatial resolution contain continuous, hydrologically sound elevation data created using void-filling algorithms. While SRTM has the advantage of providing near-global elevation data, the slope-induced errors characteristic of InSAR data [38] make SRTM unsuitable for glacier change detection at small time scales and over small glaciers. Furthermore, the acquisition month of SRTM overlapped with the accumulation season in the mid-latitudes and the outer tropics, and SRTM-derived elevations over glaciers may be over-estimated. Alternatively, DEMs derived from SPOT5, ASTER, CORONA or ALOS PRISM may be used in mass balance studies. Several studies [76-78] used SPOT5-derived DEMs for glacier studies with good results, but currently, the high cost of the SPOT5 imagery limits its use over larger areas. Older CORONA KH-4, KH 4A and KH 4B systems were also equipped with a forward looking and aft-looking camera, providing high resolution stereo imagery (< 8m) suitable for DEM generation [79]. However, the use or CORONA DEMs for glacier mass balance applications has been quite limited so far because of complicated image geometry and flight parameters, especially in rugged terrain [79].

ASTER-derived DEMs with medium spatial resolution (30m) constructed using the Silcast software are available as routine products at low costs from the LP DAAC. DEMs can also be constructed by the user using stereo-correlation procedures available in various software packages: PCI Geomatica 9.0 Orthoengine, ENVI, and Leica Photogrammetry Suite. Several studies have been undertaken to provide accuracy assessments of the ASTER-derived DEMs for glaciologic applications, for example [80-87]. Ground control points (GCPs) acquired in the field concomitantly with the ASTER scenes are needed to produce “absolute” DEMs, where locations are fitted to the UTM coordinate system and elevations are referenced to mean sea level [87]. GCPs needed for generating absolute ASTER DEMs may also be obtained from other sources, e.g. detailed topographic maps or orthorectified images. However, in politically sensitive areas, such data are either restricted, or the accuracy of the topographic maps is not known (see section 4.2). A promising solution may be the ASTER Global Topographic Map (ASTGTM), currently in production using the Silcast software. ASTGTM is expected to be released in 2008 (H. Fujisada, Sensor Information Laboratory Corp., Japan, personal communication).

### 3.3. Volume-area scaling techniques for mass balance estimations

When glacier outlines are available, ice volume can be inferred from the glacier area using the scaling relationships developed empirically by [55, 88, 89]. The scaling theory states that glacier volume (V), area (A) and length (L) are related by power laws such as A = L^α^ and V = A^β^, where and α and β are coefficients that are determined empirically. Bahr et al. [54] found β to be 1.36 for valley glaciers and 1.25 for ice sheets, based on field observations. The change in volume can also be estimated directly from changes in length using a new approach proposed by [90]:

$$\frac{V}{V_{\text{ref}}}={\left(\frac{L}{L_{\text{ref}}}\right)}^{\eta }$$

where V_ref_ and L_ref_ are reference volume and length respectively, and η is a scaling coefficient taken to be 1.4 - 1.5 for glaciers where there is no change in width, and 2.4 - 2.5 for ice caps. They recommend using an exponent of 2.0 to 2.1 for a wider range of glacier geometries, which is in agreement with coefficients found by Bahr et al. [54] based on a sample of 300 glaciers. Volume estimates determined from scaling relationships are converted to specific mass balance assuming a value for the density of the material lost or gained. In the ablation zone, a density of ice of 900 kg/m^3^ is usually considered for the conversion of volume changes to mass balance. In the accumulation area, however, the material gained or lost can range from ice (900 kg/m^3^) to firn (550-600 km/m^3^) [26]. However, most studies, for example [1, 78] assumed a constant density of ice of 900 kg/m^3^ for both the accumulation and ablation zone. This may introduce errors in the mass balance calculations, and subsequent sea-level estimates.

The volume/area scaling approach has the advantage that it is fast, convenient, and may be considered an acceptable approach for very large samples of glaciers and in cases where only 2-D information is available. However, there are several disadvantages of this method, related to local climate effects, the continentality of the climate, and the presence of debris on glacier tongues, which are not accounted for by this method [88]. Furthermore, the scaling relationships are very sensitive to the choice of parameter η. The main limitation in using these scaling relationships for assessment of glacier volumes and mass balance is that they assume steady state conditions among climate, ice flow and ice geometry, which is unrealistic. An alternate method is to use digital elevation data to extract three-dimensional glacier information that may improve the estimates in ice volume, as suggested by [91].

### 3.4. The remote sensing geodetic method

In absence of direct field measurements, mass balance can be estimated using an indirect method (“geodetic method”) which consists in measuring elevation changes over time (δh/δt) from various DEMs constructed over the glacier surface. Elevations from older DEMs, often constructed from historical topographic information, are subtracted from more recent DEMs constructed from remote-sensing imagery such as ASTER, SRTM or SPOT5, either on a pixel-by-pixel basis or as average elevation change to obtain difference maps. If elevation changes are computed pixel by pixel, the elevation differences (δh/δt) are multiplied by the pixel area to give the volumetric changes per pixel (δV/δt). If elevation differences are computed over the whole glacier surface, the average elevation change is multiplied with the glacier area to obtain the overall change in volume. The volume change is translated into mass balance change by multiplication with the density of glacier/firn (measured or estimated) as described above (section 3.3). Ideally, estimates of the vertical motion of underlying ground associated with isostatic rebound need to be included in the calculations of δh/δt, especially if the method is applied over ice sheets, as in [2]. This method yields the changes in the average mass balance expressed as meters water equivalent over the time period considered. The geodetic approach was used in several studies on the basis of historical topographic maps and DEMs derived from SPOT imagery [76, 78], SRTM [18, 92], ASTER [93, 94], laser altimetry [1] or a combination of optical imagery (SPOT HRV, Landsat TM and ASTER) and SAR (ERS, Radarsat) [95]. Several new studies used high resolution DEMs derived from ALOS PRISM and Corona [41, 96] to estimate mass balances with the geodetic method. Studies conducted in the French Alps showed a good correlation between mass balance values derived from the geodetic method and ground data [97], as well as a good correlation with mass balance reconstructions from meteorological data [98]. Another study [99] compared mass balance estimates using the geodetic, glaciologic and hydrological methods for the Tuyuksu glacier region in the northern Tien Shan, Central Asia. They found a good agreement between the geodetic method (−12.6 m w.eq.) and field glaciological measurements (−16.8 m w.eq.), and attributed the small discrepancies to errors in the field measurements.

The remote sensing geodetic method can be to validate other methods of mass balance estimations, with the advantage of being fast and easy to apply. It is however limited to estimations of changes in mass balance at decadal scales. The accuracy of the geodetic-based mass balance estimations is highly dependent on: 1) the interpolation method used to derive a DEM from digitized contours or GPS measurements [92]; 2) errors introduced by any change in spatial resolution (downscaling or upscaling) used to match the various DEMs; 3) biases inherent in the remote sensing-derived DEMs, such as elevation and slope biases [81, 84, 100]; 4) assumptions about the density of the lost or gained material. Errors in the source DEMs propagate with each arithmetic operation performed, and may introduce large errors in the output mass balance estimations, necessitating a careful evaluation or validation. Due to these large uncertainties, currently the geodetic method should only be applied for estimating changes in glacier surface and mass balance at decadal or longer time scales [52, 99].

### 3.5 AAR-ELA methods for mass balance estimations

At regional scales, mass balance can be inferred from two parameters related to a glacier's mass balance: AAR and ELA, derived from field measurements or satellite imagery. Various methods have been proposed in recent literature: i) the AAR/ELA method developed by [28]; ii) the “template” method developed by [101, 102] and iii) the ELA method for mass balance time series developed by [97, 98]. All three methods rely on the assumptions that: a) under steady state conditions, the accumulation area of a glacier (the area above the ELA) occupies a fixed percent of its total area [103] and b) the elevation of the transient snowline (SLA) at the end of the ablation season coincides with the yearly ELA [50]. The yearly ELA of a glacier can be extracted using band ratios in the visible wavelengths. The “dirty” ice or debris-covered glacier ice in the ablation zone has a lower albedo (α = 0.15 − 0.2) and is therefore less reflective than the fresh snow in the accumulation zone (α = 0.85) [26]. This difference in the reflectivity of the glacier surface in the accumulation versus the ablation zone is used to delineate the transition between the two zones, and the yearly ELA altitude is easily extracted from a DEM. The yearly AAR for a given glacier is easily determined from the ELA and glacier area delineated from satellite images (section 3.1) by calculating the area above the ELA and below the ELA. The AAR value for a given glacier varies from year to year depending on changes in its mass balance.

**Figure 1.**
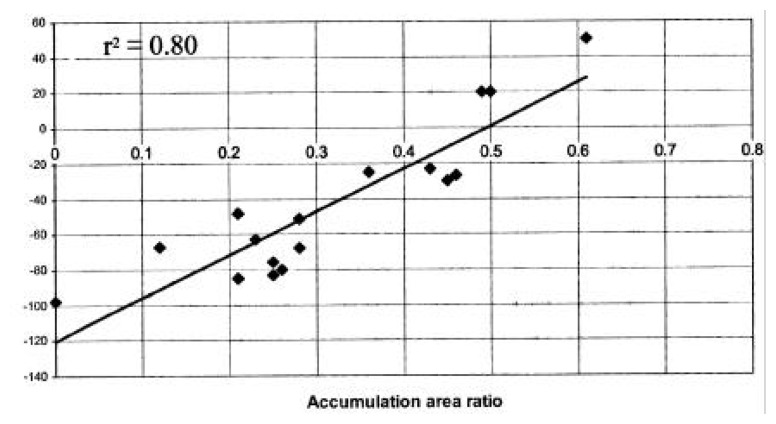
An example of the relationship between accumulation area ratio and mass balance, used to derive the steady-state AAR for Shaune Garang and Gor Garang glaciers [105].

The AAR/ELA method described in detail in [28] focuses on finding a relationship between AAR and mass balance. Then the *steady-state* AAR (the value at which the glacier is in equilibrium with the climate) can be established for a particular glacier or glaciers within one climatic region [28]. For individual glaciers, the method involves the following steps: 1) compiling field-based mass balance measurements (b_n_) and AAR for individual glaciers in a region; 2) plotting bn vs. AAR for each glacier, and finding the regression lines of the form:

(1) Bn=a*AAR+b

where Bn is the specific mass balance in water equivalent (m) and AAR is the accumulation area ratio, as shown in Fig. 1; 3) obtaining the value of AAR for which mass balance is zero from eqn.1 - this yields the *steady-state* AAR (AAR_0_), the value at which a glacier is in equilibrium with the climate. When the AAR method is applied for several glaciers in the same climatic zone, a single regression line is plotted, from which the regional AAR_0_ is obtained. For example, [28] used Landsat imagery from several glaciers in the Western Himalaya for different years, and found a generalized value of AAR_0_ of 0.44 for the Western Himalaya. This is different than the AAR_0_ of 0.67 typical of alpine glaciers [26] or an AAR_0_ of 0.82 for tropical glaciers [104]. These differences in the steady-state AAR values show the need for applying this method for each region separately.

The “template method” developed by [101, 102] is a variation of the AAR method described above, and is described in detail by [106]. The main assumption underlying this method is that within the same climatologic region, variations in mass balance are due primarily due to topographic and elevation effects, reflected in the area-altitude distribution (glacier's hypsography). The data needed are the glacier area and the ELA derived from satellite imagery, and a DEM from which the glacier hypsography curve is extracted. The hypsographic curve allows the AAR to vary with ELA according to the relationship [106]:

(2) $$\text{AAR}(\text{ELA})=\frac{1}{A_{\text{tot}}}\int_{Z_{\text{top}}}^{\text{ELA}}s(z)dz,$$

where A_tot_ is the glacier area, and s(z)dz is the change in glacier area as a function of elevation (z). The annual mass balance of the glacier, b_n_ is considered to be a linear function of AAR:

(3) $${\text{B}}_{\text{n}}={\text{a}}_{1}*(\text{AAR}(\text{ELA})+{\text{a}}_{2})$$

where the constants a_1_ and a_2_ are determined empirically from field measurements. The strength of the template method is that it allows predicting the response of a glacier's annual mass balance to ELA variations. Furthermore, it uses multi-year field measurements from one glacier or several glaciers to find b_n_ as a linear function of AAR. Thus, the linear relationship established from one glacier can be used to estimate the mass balance for unsurveyed glaciers in the same climatic area. While this method allows for changes in a glacier's hypsography, the assumption of linear b_n_ vs. AAR relationship needs to be carefully evaluated before this method is applied. The template method was applied to estimate the mass balance for the entire Ak-Shirak range in the Tien Shan, and to derive a change in volume of the glacier system for a given ELA [106].

The “ELA method” proposed by [97, 98] focuses on estimating glacier mass balance from the late summer position of the snowline on satellite images. This method is different from the AAR/ELA methods described above, as it is not based on a statistical (empirical) relationship. The ELA method involves the following steps: 1) identifying the snowline on images recorded at the end of the ablation season for each year, which can be considered as the equilibrium line for temperate glaciers [26]; 2) extracting the altitude of the snowline using a DEM, taken to be the ELA of the glacier (ELA_i_) 3) computing the total volume lost of the glacier over the studied period using the geodetic method and inferring the mean annual mass balance over the time period as described in section 3.4.; 4) calculating the steady state ELA over the time period (ELA_0_), using the equation:

$$ELA_{0}=\frac{1}{n}\sum_{i=1}^{n}ELA_{i}+\frac{\bar{B}}{\frac{\partial b}{\partial z}},$$

where *B̅* is the mean glacier-wide mass balance over the time period, determined either from field measurements using the glaciologic method, or from remote sensing using the geodetic method (section 3.4;, and *∂b*/*∂z* is the mass balance gradient at the ELA [97], and 5) calculating the glacier annual mass balance b(t) using the equation:

$$b(t)=(ELA_{0}-ELA_{i})\frac{\partial b}{\partial z}$$

This method requires an assumption about the mass balance gradient, *∂b*/*∂z* in the vicinity of the ELA. For glaciers in the Alps, [97, 98] assumed a linear relationship between mass balance and ELA,with *∂b*/*∂z* fixed at 0.78m per 100m. Similarly, [30] found a *∂b*/*∂z* value of 0.69m per 100m for the Chhota Shigri glacier in the Western Himalaya based on four years of mass balance measurements. The assumption of a linear mass balance gradient holds if there is no debris cover on the glacier tongue. In their study, [97] found this gradient to be valid at a regional scale with homogenous climate conditions. Rabatel et al. [98] showed that the vertical gradient derived from field measurements on a single glacier can be used to estimate mass balance for other glaciers of the same massif, in good agreement with field measurements. The strength of the ELA method consists in the ability to apply in-situ measurements from one or more glaciers to a glacier or groups of glaciers that don't have field measurements, just like the AAR/ELA and the template method described above [98]. This method is robust if the mass balance gradient is linear, as is generally the case of clean (debris-free) glaciers. Complications arise when debris is present. Debris cover influences the surface energy balance, changing the ablation component, and thus affecting the mass-balance gradient [107, 108]. The question remains, however, whether the assumptions of invariant mass balance gradient and invariant hypsography are valid for wide application of this method.

## 4. Case study: Application of remote sensing methods to the Himalaya

The high Himalaya provides both interesting challenges and unique opportunities for testing the new remote sensing tools described above to estimate glacier mass balance. There is a wide variety of glacier sizes, types, dynamics, topography and debris-cover in this region. There are large gradients in climate due to the weakening in the intensity of the Indian monsoon from east to west and from south to north [44], which induces variability in the glacier cover and its response to climate forcing. The wide altitude range and the variability in debris cover make the Himalayan glaciers particularly sensitive to climate forcing [109]. Most importantly, field based measurements of mass balance in the Himalaya are sparse, and Himalayan glaciers are conspicuously absent from global mass balance records such as [21, 110]. In summary, dynamic climate and glaciology make this region an excellent choice for extensive testing of the remote sensing-derived glacial characteristics and their application to glacier change detection.

### 4.1 Himalayan glacier fluctuations

With 15% of its area covered by glaciers, the Himalaya constitutes the largest glacier system in the world outside Antarctica and Greenland [111]. The Himalayan ranges are home to some of the longest glaciers in the world: Siachen (72km), Bara Shigri (28km), Gangotri (26km), Zemu (26km), Milam (19km) and Kedarnath (14.5km) [112]. Direct observations of termini of Himalayan glaciers have shown these glaciers to be in a state of general retreat since the last century [113]. The trend has apparently accelerated in the last several decades across the Himalaya [112], with strong regional variability. Examples include Glacier AX010 (0.57 km^2^) in the Shorong Himal, which retreated 30 m from 1978 to 1989 [11]. Glacier retreat rates of up to 52 m/year were reported from the Western Indian Himalaya [14, 114] and 30 – 40m/year from the Bhutan Himalaya [115]. The retreat of Nepalese glaciers in the last three decades was documented by several studies: [11-13, 116]. This trend may be linked with observed temperature increases of 0.06°C/year in the Himalaya since the 1970s [117]. However, there may be regional differences in glacier response due to regional climate or non-climate factors such as the presence of debris cover. The effect of debris cover on the ablation component of mass balance may override climate-induced changes, but this effect is not well understood [108]. Debris-covered tongues in the Himalaya were found to be relatively stable since the last major glacier advance about 150 years ago, but the lower sections have been thinned by as much as 70m, for example Khumbu glacier in the Nepal Himalaya [118].

### 4.2 Himalayan glaciologic data

**Figure 2.**
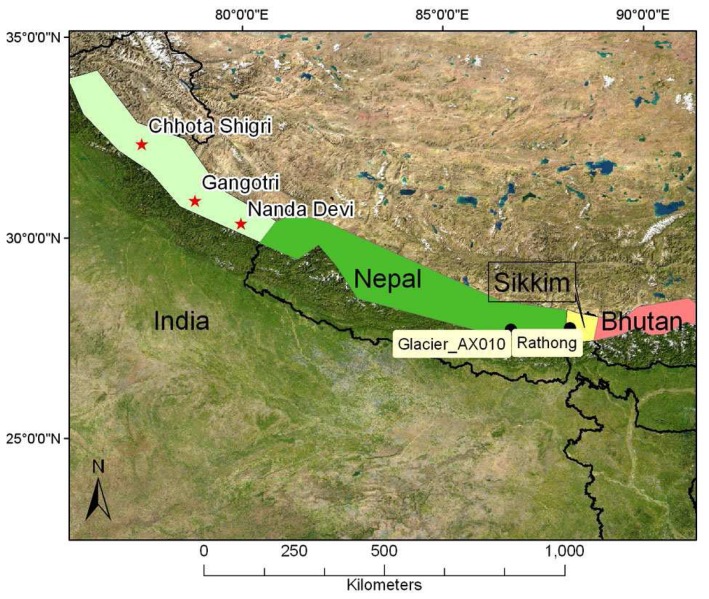
Study area showing surveyed sites during the 2006 field campaign (red stars) and additional validation sites proposed for the 2007 field campaign (black circles), shown on a MODIS mosaic.

**Figure 3.**
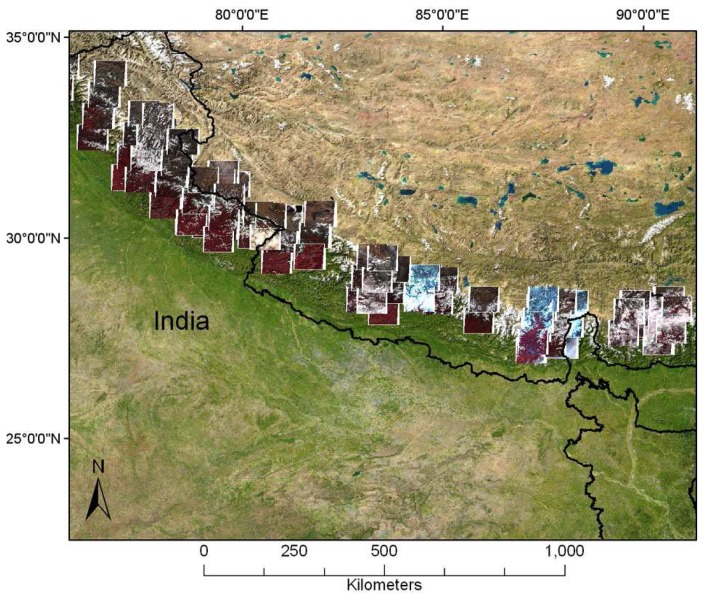
Coverage of the 2006 Data Acquisition Request (DAR) submitted by the GLIMS team, showing the 115 ASTER scenes acquired during Sept – Nov 2006.

*ASTER imagery:* 14466 ASTER scenes acquired from 2000 to 2008 over the Himalaya. Many of these were unsuitable for glaciologic analysis because of cloud-cover or low contrast over snow and ice. Starting with 2006, we submitted high priority data acquisition requests (DARs) to the ASTER Science Team, through the Global Land and Ice Monitoring from Space (GLIMS) project. The Himalayan DAR consisted of four polygons covering glacierized areas in India, Nepal, Sikkim and Bhutan (Fig. 2), and resulted in 115 new ASTER Himalaya scenes in 2006 (Fig. 3) and 216 scenes in 2007. However, starting with May 2007, the ASTER SWIR instrument has been deteriorating quickly, posing problems for future ASTER scene acquisition in the Himalayas. The ALOS sensor may provide a valuable option for future data acquisition.

*Elevation data:* DEMs derived from old topographic maps and aerial photography can be used as baseline for comparison with more recent satellite-derived DEMs. In the Indian Himalayas, such DEMs are sparse due to restrictions on aerial photographs and topographic maps at scales larger than 1: 100,000 from areas 80 km wide along the external land border and coastline. This includes Jammu and Kashmir, the eastern districts of Himachal Pradesh, the northern districts of Uttarakhand area of Uttar Pradesh, Sikkim, and the whole of northeast Indian Himalaya. There are also restrictions on export of maps, aerial photographs, and trigonometric and gravity data. At present, aerial photography is classified as top secret for the whole of India. The old British Survey of India 1:63,360 scale maps published in 1930 are no longer available, and the Government of India maps at a similar scale are also classified. The only available map series covering the entire Indian Himalaya region are the US AMS Series U502 at 1:250,000 scale, published in 1959-1963, and the Soviet 1:100,000 Military Topographic Maps. However, the accuracy and the scale of these maps are not sufficient for glaciologic applications. In the Nepal Himalaya, in contrast, access to large scale topographic data is not restricted. Nepal is covered by the new 48 sheets at the 1:50,000 scale, published by the Survey Department of His Majesty's Government of Nepal in 2001 based on 1992 aerial photography, and available for purchase in digital form from the Survey Department, National Geographic Information Infrastructure Program (NGIIP) (http://www.ngiip.gov.np).

DEMs constructed from satellite imagery (ASTER, SRTM, SPOT5, IRS-1C or CORONA) are increasingly being used for glaciologic studies in the Himalaya. DEMs from the Indian Remote Sensing Satellite (IRS-1C) exist from a few areas such as the Baspa valley in the Himachal Pradesh district of India [15, 119, 120]; however, they are not in the public domain. ASTER DEMs have been used in recent glaciologic studies in the Nepal and Bhutan Himalaya, for example [10, 79, 84]. However, the most readily available sources of remote sensing-derived elevation data over the Himalaya is currently still the SRTM DEM version 3, which may be combined with higher resolution DEMs. Several studies focused on evaluating DEMs from stereo satellite data in the Himalaya, for example [15, 84]. Berthier et al. [78] compared SRTM elevations with SPOT5–derived elevations on non-glaciated terrain and found a mean difference of 0.43m and standard deviation of 16.7 m after correction of horizontal shifts. Recently, [84] evaluated the performance of ASTER and SRTM DEMs in the Bhutan Himalaya. They reported an RMSEz of the altitudinal difference with respect to ground survey data of 11 m for ASTER DEMs and 11.3 m for SRTM DEMs. A comparison of SRTM-derived elevations with ICESAT elevation profiles in rugged relief of Central Asia showed errors to increase in complex terrain (standard deviation > 30 m) [121]. Large elevation errors were found at higher elevations in the French Alps also [100] and steeper slopes in the Tien Shan [18] and the Andes [92].

*Existing glacier inventories:* The International Center for Integrated Mountain Development (ICIMOD), Nepal, produced a comprehensive inventory of glaciers and lakes in the Himalaya for the India, Nepal, Bhutan and Pakistan from various data sources, including Landsat imagery, IRS and Survey of India maps. All the datasets are distributed freely on CD-ROMs (http://www.icimod.org). The datasets and are currently being checked for accuracy and soon ingested in the GLIMS Glacier Database. Other inventories constructed from remote sensing were derived for the Western Himalaya from IRS and Landsat TM data from the 1980s [122]. Glacier mapping has been conducted in the Parbati basin from IRS-P6 (LISS-IV sensor) and in the Baspa and Chenab basin from IRS-1D (LISS-III sensor) [123, 124]. Both basins are located in the Himachal Pradesh district in the Western Indian Himalaya. A recent inventory for the Lahaul-Spiti region in Western Indian Himalayas was constructed using manual digitization from 2002 ASTER imagery [78]. The inventory covers a glacierized area of 915 km^2^ and is available from the GLIMS Glacier Database. Glacier areas for Sikkim, derived from IRS-1A and Landsat TM data from 1987/1988 are reported in [35, 36]. Another glacier inventory for Sikkim was constructed from 1992 and 1997 IRS 1A, 1B, 1C and 1D data [125, 126]. The Nepalese and Bhutan Himalaya glacier datasets, along with methodology and results are available from ICIMOD [32, 33]. Glacier area changes from the Khumbu Himalaya (Nepal) based on 1962 Corona, 1992 Landsat TM and 2001/2005 ASTER data are reported in [79].

*Field measurements:* For the most part, mass balance measurements in the Himalaya remain sparse. Field-based mass balance measurements have been conducted since 2002 at Chhota Shigri, a benchmark glacier in the Lahaul-Spiti district of the Indian Himalaya [30]. Other field investigations were conducted by Indian teams from 1988 and 2003 at Chhota Shigri, and are reported in [123]. Some field based mass balance data exist from Gara, Gora Gorang and Neh-Nar glaciers in the Western Himalaya, in unpublished reports from the Geological Survey of India [28, 105]. Field measurements of glacier area and tongue fluctuations were also conducted at Gangotri Glacier in the Garwhal Himalaya, India, and reported in [127, 128]. Mass-balance measurements for the Glacier AX010 in Solu-Khumbu, Nepal were conducted in 1978 - 1979, with only limited observations since that time [11, 31]. The mass balance of Langtang Glacier in Nepal was estimated from temperature records (1969 – 1997), precipitation records (1987 – 1997) from low altitudes (Kathmandu, 60 km away) and the area-altitude distribution of the glacier [29].

*Climate data:* Precipitation, temperature and hydrological data are needed for input to energy balance models, and to correlate observed changes in glacier area from satellite data with climate trends. For the Indian Himalayas, such measurements are scarce and difficult to obtain. Manual measurements have been conducted at lower elevations by the Indian Meteorological Department, but these data are restricted. For Nepal, the Department of Hydrology and Meteorology (http://www.dhm.gov.np) maintains nation-wide networks of 337 precipitation stations, 154 hydrometric stations, 68 climatic stations, 22 agro-meteorological stations, 9 synoptic stations and 4 aero-synoptic stations. Temperature, precipitation, discharge, sediment load, water quality and other parameters are available to users through published reports, bulletins, or by purchase in digital form. A few climate stations exist at high-altitudes in the Langtang area [129]. An Automatic Weather Station (AWS) was installed in 1994 at Syamboche village in the Solu-Khumbu district, at an altitude of 3,833 m [130, 131]. In addition, climate data are acquired at The Pyramid International Laboratory-Observatory, located at 5,050 m.a.s.l in the Khumbu region (Nepal), at the base of the Nepalese side of Everest. In general, however, climate data from higher altitude stations (> 2,000 m) are limited throughout the Himalaya.

### 4.3 Previous remote sensing mass balance estimations in the Himalaya

Progress on applying the remote sensing methods for mass balance of the Himalayan glaciers has been slow due to the lack of accurate elevation data. The increased availability of recent DEMs from stereo imagery provide some opportunities to derive mass balance at shorter time scales in the Himalaya, when careful error assessments are done. For example, [78] estimated mass balance changes in the Indian Himalaya using DEMs from 2004 SPOT5 and 2000 SRTM elevation data. They found a significant thinning of the glacier surface of -8 to -10 m at lower elevations including debris-cover tongues, and less thinning in the upper parts of the glaciers (-2 m). The mass balance calculations were within the range of field-based mass balance measurements from the same period in the Lahul-Spiti region [30], which shows promise for the application of geodetic methods for the Himalayas. Studies in other areas found a strong dependency of glacier elevation changes with altitude, with the largest glacial changes (thinning) at glacier termini and less changes at higher elevations [1, 78, 92]

**Figure 4.**
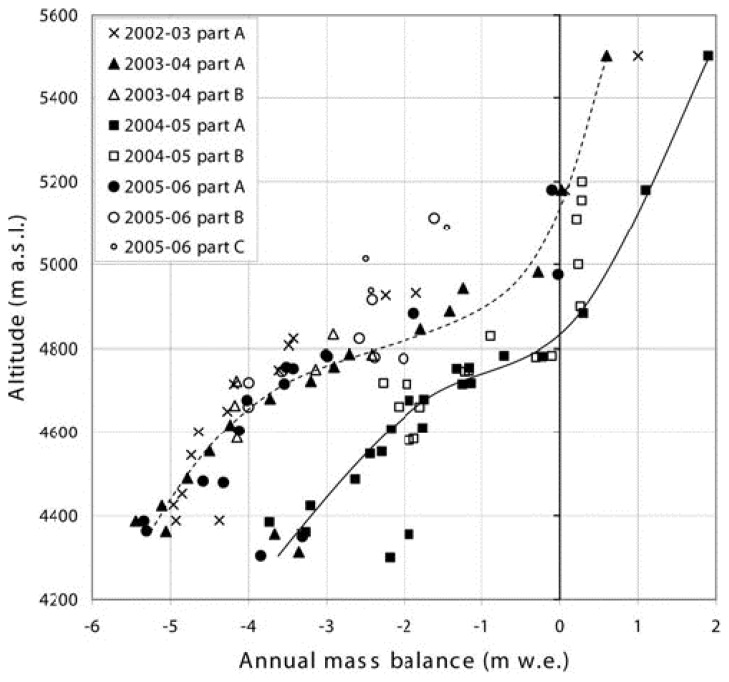
Mass-balance at Chhota Shigri glacier as a function of altitude, derived from 4 years of field-based measurements on various glacier tributaries. Courtesy of IRD France, reproduced from [30].

The ELA-AAR method proposed by [28] was applied sparingly so far in the Himalaya due to limited field-based mass balance measurements needed to infer the b_n_-ELA relationship. Based on field measurements from several glaciers in the Western Himalaya, [35, 105] proposed a steady-state AAR for the Western Himalaya of 0.44 based on field mass balance measurements. The bn vs. ELA relationship was used at Chhota Shigri glacier in Lahul-Spiti in the Western Indian Himalaya [30] to infer an average ELA and AAR (Fig. 4). The study reported a strong negative mass balance of up to -1.4 m water equivalent for the period 2002 – 2006. The average ELA was ∼ 5180 m and the AAR was 0.3 for all years except 2004/2005 when the mass balance was positive. Continued mass balance measurements are key to establish a relationship between mass balance and AAR and to test the ELA/AAR methods for other areas of the Himalaya.

### 4.4 Progress on mapping of Himalayan glaciers using remote sensing

**Figure 5.**
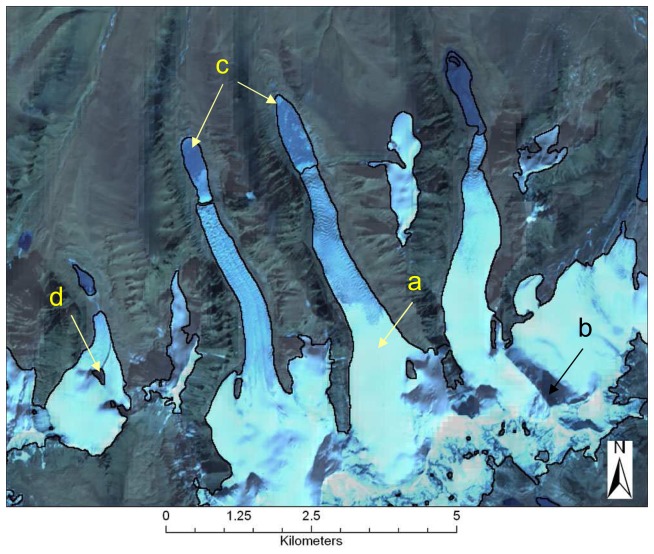
Results of the classification algorithm for clean ice in Northern Sikkim, from 2001 ASTER imagery. Arrows point to: a) clean ice correctly classified; b) shadowed glacier correctly classified; c) pro-glacial lakes mis-classified as glacier; d) internal rock correctly delineated.

Most of the glacier mapping in the Himalaya has been conducted using Landsat TM and/or IRS data, for example [36, 120, 124]. Some studies used false color composites (FCC) to delineate the extent of clean (debris-free ice). For example, [36] used a FCC from IRS 1A and Landsat TM in the Sikkim area to delineate glacier boundaries, accumulation and ablation areas, ELA and glacier lakes. In the Lahul Spiti area (Western Indian Himalayas), [78] digitized glacier boundaries manually from 2002 ASTER data, with an estimated accuracy of ±2 pixels (30 m). Other studies, for example [132] used band ratio techniques (NDSI) to map glacier features from Landsat/IRS data. Of the available band ratio algorithms, they found NDSI to perform the best in delineating snow/ice versus bare terrain. However, a recent study [79] tested various image ratios and supervised classification techniques for the Khumbu Himalaya and found that they overestimate the accumulation zone due to low contrast in the upper parts of the glaciers. If high contrast images are available, such as ASTER scenes acquired with GLIMS gains, the NDSI algorithms may still provide optimal results. An example of mapping of clean glaciers in the Sikkim Himalaya (India) derived from an ASTER scene from Nov 27, 2001 is shown in Fig. 5. For illustration purposes, we delineated clean ice and snow using the NDSI algorithm from ASTER bands 1 and 4. The NDSI method correctly classified shadowed ice as glacier, and masked low clouds, but failed to distinguish frozen pro-glacial lakes from glacier ice (Fig. 5).

Automatic mapping of glacier lakes in the Himalayas was addressed only in a few studies. One study [67] classified glacier lakes in the Everest area of Nepal based on varying turbidity as indicated by the lake color in visible/near infrared ASTER bands 1-3. Zhang et al. [68] developed a high accuracy sub-pixel mapping algorithm for lake cover monitoring in the Tibetan Plateau based on linear spectral unmixing techniques using ASTER imagery. However, in the Himalaya, automatic mapping of glaciers is complicated primarily by the presence of debris cover on glacier tongues, which confounds the image classification techniques. Most studies delineated the debris cover manually using FCC maps and topographic information. For Sikkim, one study [36] delineated the debris cover area using images from August and September. At that time of the year, grass on the terminal and lateral moraine gives a red tone on the FCC, helping to delineate the debris cover area. Other studies [39, 73, 133, 134] proposed semi-automatic methods for the delineation of debris-covered glaciers using satellite imagery combined with terrain information. Bolch et al. [134] combined morphologic features (terrain slope and curvature) extracted from an ASTER DEM with thermal information in a supervised classification approach in the Everest area of Nepal, with an accuracy of 5% when compared to manually digitized polygons. The use of ASTER thermal bands alone for debris cover mapping in the Himalaya has also been exploited in a few studies [73, 135, 136]. For example, Suzuki et al. [136] used ASTER thermal IR data to derive spatial distributions of thermal resistances on debris-covered glaciers, to study the evolution of glacier lakes in the Bhutan Himalayas. While these approaches hold promise for glacier mapping in the Himalaya, field validation of the automatic algorithms is an ongoing challenge. Problems arise in marking the limit between debris covered ice and non-ice debris, and distinguishing between active glacier and stagnant ice area [134]. Supra-glacial ponds on the glacier tongue covered by debris may be used as an indicator for the presence of ice when possible.

## 5. Conclusions

Remote sensing offers promise for glacier monitoring in areas lacking traditional glaciologic methods. This paper reviewed key advances in the use of optical remote sensing for mass balance of mountain glaciers, with an emphasis on current algorithms and their limitations in the Himalayas. Ongoing challenges in applying the proposed methods at large scales remain, such as: 1) the lack of standardized image analysis methods for delineation of debris-covered ice; 2) limited field validation data (GPS measurements and specific mass balance measurements); 3) lack of accurate elevation data for remote glacierized areas; and 4) algorithms for automatically discerning debris-covered ice from non-ice areas with debris. In the Himalaya in particular, further limitations are posed by: i) restrictions imposed on use and export of topographic maps along with trigonometric and gravity data needed to interpret aerial photography; ii) difficulty of acquiring cloud-free ASTER scenes at the end of the ablation season; and iii) limited field-based mass balance measurements with long-term record for validation of the ELA/AAR method. Continued acquisition of satellite scenes (ASTER, ALOS) and ground control points are needed to cover the entire Himalaya, both spatially and temporally, with high-quality data suitable for glacier analysis. On-going work focuses on field-validating the proposed algorithms for debris-cover delineation and evaluating different software packages to generate DEMs from ASTER data. With careful evaluation and validation, these new remote sensing techniques will help advance our understanding of the response of both clean and debris-covered glaciers to climate forcings worldwide, and will help to better estimate future water resources, glacial hazards and the glacier contribution to sea-level rise.
